# Tracking the reach of COVID-19 kin loss with a bereavement multiplier applied to the United States

**DOI:** 10.1073/pnas.2007476117

**Published:** 2020-07-10

**Authors:** Ashton M. Verdery, Emily Smith-Greenaway, Rachel Margolis, Jonathan Daw

**Affiliations:** ^a^Department of Sociology and Criminology, Pennsylvania State University, University Park, PA 16802;; ^b^Department of Sociology, University of Southern California, Los Angeles, CA 90089;; ^c^Department of Sociology, University of Western Ontario, London, ON N6A 5C2, Canada

**Keywords:** COVID-19, demography, mortality, bereavement, social support

## Abstract

The coronavirus disease 2019 (COVID-19) pandemic has led to a large increase in mortality in the United States and around the world, leaving many grieving the sudden loss of family members. We created an indicator—the COVID-19 bereavement multiplier—that estimates the average number of individuals who will experience the death of a close relative (defined as a grandparent, parent, sibling, spouse, or child) for each COVID-19 death. Using demographic microsimulation-based estimates of kinship networks in the United States, the clear age gradient in COVID-19 mortality seen across contexts, and several hypothetical infection prevalence scenarios, we estimate COVID-19 bereavement multipliers for White and Black individuals in the United States. Our analysis shows that for every COVID-19 death, approximately nine surviving Americans will lose a grandparent, parent, sibling, spouse, or child. These estimates imply, for example, that if 190,000 Americans die from COVID-19, as some models project, then ∼1.7 million will experience the death of a close relative. We demonstrate that our estimates of the bereavement multiplier are stable across epidemiological realities, including infection scenarios, total number of deaths, and the distribution of deaths, which means researchers can estimate the bereavement burden over the course of the epidemic in lockstep with rising death tolls. In addition, we provide estimates of bereavement multipliers by age group, types of kin loss, and race to illuminate prospective disparities. The bereavement multiplier is a useful indicator for tracking COVID-19’s multiplicative impact as it reverberates across American families and can be tailored to other causes of death.

## Significance

COVID-19 has created a mortality shock throughout the world, and it may yield a second wave of population health concerns tied to bereavement and social support reductions. We created the COVID-19 bereavement multiplier, an indicator that clarifies one downstream impact of COVID-19 mortality and can be applied to different epidemiological projections of death counts: How many people are at risk for losing a grandparent, parent, sibling, spouse, or child for each COVID-19 death. In the United States, we estimate that on average, under diverse epidemiological circumstances, every death from COVID-19 will leave approximately nine bereaved. Studying how acute mortality crises reverberate through a population in the form of bereavement multipliers expands understandings of the social impacts of health crises.

The novel coronavirus outbreak has led to an abrupt rise in mortality throughout the world and already left many grieving the sudden loss of relatives from a new disease, coronavirus disease 2019 (COVID-19). It remains unknown, however, how many people have experienced the death of a close relative, and how many in total will over the course of the pandemic. In this paper, we generate and apply an indicator to the United States—the COVID-19 bereavement multiplier—that approximates how many Americans will be left grieving the death of a grandparent, parent, sibling, spouse, or child for each COVID-19–associated death under a variety of different infection-prevalence scenarios. Drawing on the age pattern of COVID-19 mortality in different contexts and kinship networks in the United States estimated from demographic microsimulation (1, 2), we demonstrate that the COVID-19 bereavement multiplier is highly stable to epidemiological variation (e.g., infection rate, total deaths, and distribution of deaths), meaning that the results are constant under a range of potential epidemic trajectories.

Studying the burden of COVID-19 mortality from the perspective of bereaved kin is important for two reasons. First, these estimates will offer a sense of the reach of COVID-19 deaths as they intimately affect the lives of the surviving population. Most deceased individuals are a close relation to numerous others, leading each death to be experienced by several others in their kinship network (3). For example, a 65-y-old man’s death could leave a spouse, two surviving siblings, two children, and four grandchildren bereaved. Kin represent some of the most important social ties (4–6). Having a family member recently die is tied to an elevated risk of physical and mental health decline (7–11) and broader adverse implications for individuals’ social, economic, and relationship well-being (12–15). Quantifying the average bereavement burden associated with each death can help to clarify the size of a potential second wave of population health issues tied to bereavement. Moreover, in the United States, generating race-specific estimates of the bereavement burden can clarify the longer-term family consequences of disparities in COVID-19 mortality (16).

Second, characterizing the scale of COVID-19–related kin death will also offer a new perspective of how the COVID-19 epidemic affects those at different ages. The risks of severe symptoms, hospitalization, and mortality due to COVID-19 have a clear age pattern, with lower risks among children and young adults and steeply increasing risks for older adults (17). This has led to the general understanding that older adults are especially vulnerable to COVID-19. However, the age pattern of bereavement is unlikely to mirror the age pattern of mortality; instead, it may have a completely different age gradient due to inter- and intragenerational relationship structures. Understanding the age-gradient of those who experience family bereavement due to COVID-19 is likely to clarify broader, downstream challenges. Older adults may experience a double burden of COVID-19: Not only are they most vulnerable to succumbing to COVID-19 if infected, but they may also be at disproportionate risk of losing a close relation, especially a spouse or sibling. However, younger individuals are likely to be at comparably high risk of losing parents and grandparents, the subset of the population with the highest documented COVID-19 fatality rates. Tracking these differences will illuminate some of the broader population health challenges that COVID-19–associated deaths will leave in their wake, and the potential for them to act as future sources of disparities among youth (18–20).

Although various types of kin loss at all ages are detrimental (7, 10, 21–24), for adolescents and younger adults, parental and grandparental death correspond with particularly adverse outcomes (9, 10, 19, 25–29). The negative effects of losing a parent or grandparent may be due to more limited attention of direct caregivers (25) and severed or weakened connections to other family members (30). In addition to the risk of poor mental health associated with grief and bereavement, losing kin who represent key sources of social support can also fundamentally alter youths’ access to economic security and, in turn, the success and timing of their transition to adulthood. In the United States, racial inequality in kin loss from COVID-19 may pile atop and exacerbate existing racial disparities in family member loss and access to social support among youth (6, 19). Explicitly tracking how youth are affected by COVID-19 mortality, and whether this burden is heavier among some youth populations (e.g., Black youth in America), will clarify the extent to which the epidemic could shape future life-course trajectories.

## Introducing an Indicator: COVID-19 Bereavement Multiplier

Even with the clear need to identify the extent to which COVID-19 mortality will correspond with kin loss, doing so is challenging, particularly in the midst of an ongoing mortality crisis. An expanding body of literature uses survey data to quantify how different mortality conditions result in experiences of kin death among surviving family members. For example, recent survey-based research shows how all-cause mortality can translate into unequal burdens of familial loss (19), including specific types of loss from the perspective of parents (3), siblings (9), and children (31). Of course, survey data on family deaths can suffer recall biases associated with social network data collection (32, 33) and, unfortunately, survey data on the experience of family death are censored during an ongoing crisis given that some who are currently unaffected will go on to later be affected. Although such data will eventually become available in nationally representative studies, and although there are ongoing public opinion polls that can help track in-the-moment estimates of this phenomenon, neither source can offer a contemporaneous assessment of the anticipated total losses from an evolving health crisis like COVID-19.

Beyond survey methods, scholars have long-recognized the potential to combine information on kin structures and mortality to indirectly estimate mortality conditions (34), and they have recently leveraged these insights to develop a sense of the burden of various types of familial loss (35, 36). Such an approach is also well-suited to generating bereavement estimates tied to specific causes of death, although we are not aware of any effort to do so. Of course, indirect estimation approaches still require some survey data, specifically data on kinship structures. Such data are rarely collected in the United States or abroad. Social science surveys that do include questions about the number and vital status of kin typically ask respondents to report only on their current household members, and rarely ask about their broader kin network (and those that do tend to ask a limited set of questions about a limited number of relations, disallowing a complete analysis of kin loss).

Given the lack of available survey data, here we identify the COVID-19 bereavement burden in the United States by drawing on a computational analysis of COVID-19 mortality in the kinship networks of White and Black Americans estimated from demographic microsimulation (1). Simulated kinship networks enable us to identify how many of each type of kin are alive at different ages, so that we can estimate how many close family members might experience the unexpected loss of kin due to COVID-19, overall, and by race, age group, and kin type.

Rather than projecting the total number of individuals bereaved by COVID-19 under diverse epidemiological scenarios, we go one step further to generate an additional indicator: A bereavement multiplier showing the ratio of the number of relatives who will be bereaved by each COVID-19 death. Rather than offering in-the-moment estimates of how many are currently bereaved from COVID-19 (estimates that will offer an incomplete picture of the disease’s social implications as long as its trajectory in terms of death counts remains uncertain) by extracting the multiplier and demonstrating its stability under diverse epidemiological trajectories, we offer an estimation approach better suited to understanding long-run social ramifications, one that ties expected rises in bereavement with corresponding increases in COVID-19 mortality. A multiplier approach is also well-equipped to demonstrate how inequity in mortality due to a specific cause of death, such as COVID-19, translates into further inequality in the extent of family loss. That is, with race-disaggregated data on the total number of COVID-19 deaths, it is possible to estimate the race-specific estimates of the total number of bereaved individuals using simple multiplication.

## Results

We drew on previously analyzed estimates of White and Black American’s kinship networks that used demographic microsimulation parameterized with age-, sex-, and race-specific demographic rates from the historical record or recent Census Bureau national projections to create complete kinship networks within race-groups (1, 2). Prior work has described how these data reflect key features of American kinship networks (1, 2), and we further evaluated their fidelity to empirical estimates of kinship networks from the Panel Study of Income Dynamics (37), specifically the mean numbers of connections between kin of different ages (*SI Appendix*, Figs. S11–S14). Upon these networks, we simulated infection and fatality scenarios (see Approach, Methods, Data, and Measures for more details).

Available data shows the age pattern of COVID-19 mortality collected in a variety of contexts with raw case fatality ratios (17), which are the number of COVID-19–attributed deaths at a given age divided by the number of confirmed COVID-19 infections among people of that age. Unfortunately, because of the ongoing nature of the pandemic and the limitations of mortality estimation and reporting, raw case fatality ratios are necessarily incomplete and do not account for censoring or biases in the accurate enumeration of deaths or diagnosis of cases; even so, these estimates confirm a consistent age-gradient in COVID-19 lethality.

Table 1 presents estimates of the COVID-19 bereavement multiplier, calculated as the ratio of the number of people who experience the loss of kin (grandparent, parents, sibling, spouse, or child) for each COVID-19 death. We calculated these multipliers using recent estimates of infection fatality ratios (the proportion of those infected who die) by age that adjust for censoring, misattribution, and nonreporting using data from Wuhan, China (38). Although we estimated using three simulated infection prevalence scenarios, wherein 10%, 20%, or 40% of the population is infected, distributed uniformly at random, here we show models from the 20% infection prevalence scenario only because the different infection prevalence scenarios yield stable estimates (*SI Appendix*, Table S1). In *SI Appendix* we also test numerous alternative models, including different infection fatality ratio parameters and nonuniformly distributed infection prevalence (*SI Appendix*, Table S1). Together, these estimates clarify the stability of the bereavement multiplier across race groups and showcase its stability under diverse infection scenarios.

**Table 1.** Bereavement multipliers for select kin, population combined, and race disaggregated results: Number of people who have lost one or more of these kin types

	Bereavement multiplier*: Number of people who would lose the named kin for each death^†^					
Population of interest^‡^	Any type^§^	Grandparent	Parent	Sibling	Spouse	Child
White and Black combined	8.91	4.01	2.15	2.04	0.46	0.20
	[7.89, 9.87]	[3.37, 4.71]	[1.81, 2.63]	[1.75, 2.4]	[0.33, 0.61]	[0.13, 0.32]
White only	8.86	3.95	2.12	2.07	0.47	0.20
	[7.77, 9.98]	[3.26, 4.77]	[1.75, 2.65]	[1.74, 2.47]	[0.33, 0.64]	[0.12, 0.34]
Black only	9.18	4.43	2.36	1.80	0.37	0.19
	[8.07, 10.27]	[3.70, 5.20]	[1.96, 2.85]	[1.52, 2.11]	[0.26, 0.50]	[0.11, 0.31]

* A bereavement multiplier of 4 in the grandparent column means that if 100,000 people die, 400,000 grandchildren would lose at least one grandparent.

^†^ Median results in the distribution of simulated estimates; the upper and lower bounds that contain 95% of the simulated results are shown in brackets.

^‡^ All models run with 20% infection prevalence, distributed uniformly at random. See *SI Appendix*, Table S1 for alternate infection prevalence scenarios. “White only” and “Black only” refer to race-specific estimates of the multipliers under the 20% infection prevalence scenario; see *SI Appendix*, Table S1, *Section D*.

^§^ Any type includes a grandparent, parent, sibling, spouse, or child.

Table 1 shows the bereavement multiplier, including the summative multiplier across kin types, kin-specific ones, and race differences. The “any type” column in Table 1 for the combined results shows that we expect each COVID-19 death to translate into 8.91 surviving individuals having experienced the death of a grandparent, parent, sibling, spouse, or child. For example, under this scenario, for every 100,000 White and Black Americans who die due to COVID-19, ∼891,000 surviving White and Black Americans will be left grieving the death of at least one grandparent, parent, sibling, spouse, or child. In 95% of the simulated results we obtained, the multiplier estimate was between 7.89 and 9.87. We also calculated these multipliers within race-groups. For White Americans, our median race-specific multiplier estimate is 8.86, while it is 9.18 for Black Americans, both comparable to the estimates obtained for the overall population. These race-specific multipliers can help us to understand racial disparities in bereavement. That is, within the hypothetical example of 100,000 deaths among White and Black Americans, if 40% of all hypothetical deaths are of White Americans, with a bereavement multiplier of 8.86, this would result in ∼354.4 thousand White Americans having lost one close relative. And, assuming 60% of all hypothetical deaths are of Black Americans, with a bereavement multiplier of 9.18, this would mean that ∼550.8 thousand Black Americans would have lost one close relative.

Table 1 further shows most kin loss will be due to the death of grandparents, followed by the deaths of parents and siblings. The population averaged bereavement multiplier suggests the COVID-19 death toll will generate roughly four times as many surviving individuals bereaved due to the death of a grandparent, and upwards of twice as many individuals will be left grieving the death of a parent or sibling. Individuals bereaved from the death of a spouse or child are far fewer; however, they still comprise a notable proportion of the bereaved. Even race differences in the kin-specific bereavement multipliers are small, although Black Americans have slightly higher losses of some vertical kin ties (grandparents and parents) and lower losses of horizontal kin ties (spouses and siblings).

**Fig. 1.**
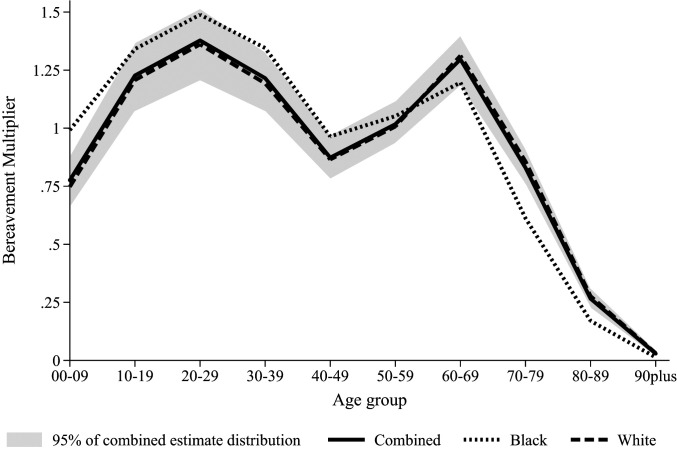
The age pattern of the bereavement burden, overall and by race: Bereavement multipliers for deaths of any type of kin by age group by different considerations of race. Note: Kin types included in the bereavement burden are grandparent, parent, sibling, spouse, and child. The areas under each curve sum to the “any kin” bereavement multipliers in Table 1. The shaded area contains 95% of the simulated distribution of combined race estimates.

Fig. 1 portrays age-specific bereavement multipliers to examine the age groups of surviving Americans who will be most affected by COVID-19 kin loss. It shows the combined results and their distribution as well as race-specific estimates, although, as noted above, they are quite comparable (see also *SI Appendix*, Table S1). Fig. 1 demonstrates that, unlike the steep age-gradient of COVID-19 mortality, the bereavement burden assumes a bimodal distribution, with youth and young adults, as well as older adults, highly affected by COVID-19 kin loss. As shown, the combined model for Fig. 1 demonstrates that for every 100,000 Americans who die to COVID-19, between 125,000 and 150,000 young people (ages 10 to 29 y) will experience a family member die; we see similar numbers among those 60 to 69 y old. Note that the areas under curves in Fig. 1 are equal to the multipliers for loss of any kin in Table 1. Race differences in this figure are subtle, but there is evidence the multiplier is larger at younger ages for Black Americans and higher at older ages for White Americans.

**Fig. 2.**
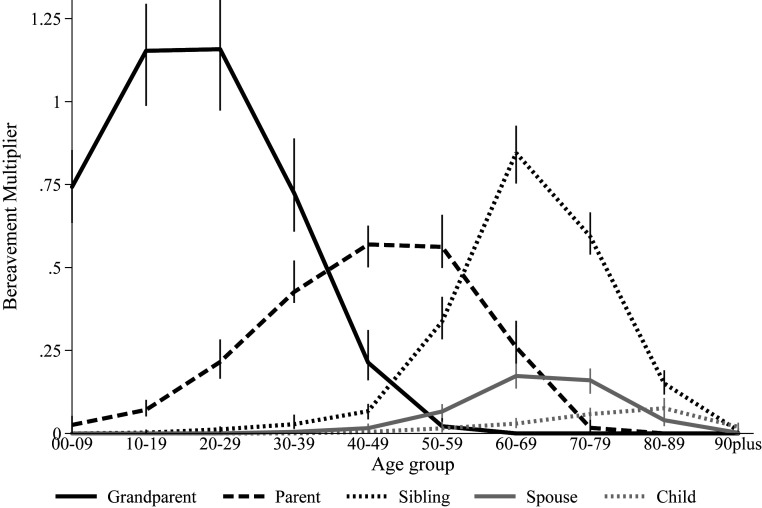
The age pattern of bereavement burden by type of kin who died. Note: The vertical bars represent 95% of the simulated distribution of estimates.

Fig. 2 further disaggregates the age gradient in kin loss to demonstrate the specific types of kin death for people of different ages for the combined population. Fig. 2 shows that, unsurprisingly, most young Americans who have a relative die will experience a grandparent’s death. Conversely, adults ages 30 to 40 y are most likely to lose a parent, whereas older adults are most likely to experience a siblings’ or spouse’s death. Fig. 2 further shows that, across all age groups, experiencing the death of a child due to COVID-19 will be rare relative to losing other kin and, when it does occur, will be concentrated among the oldest age ranges.

## Sensitivity Analyses

Supplementary analyses reinforce the value of the COVID-19 bereavement multiplier. Because the structure of both kin networks and the COVID-19 fatality are age-graded, the bereavement multipliers are highly consistent even if infection prevalence varies. The results would not, however, be consistent with different kinship networks, of course, rendering these estimates—but not the overall approach—specific to the contemporary United States. Although there are several similarities in kinship networks between the United States and other contexts, like many countries in Western Europe, there are also enough differences to leave open the question of whether these results would readily apply elsewhere. For example, recent work demonstrates that the prevalence of older adults with neither a living spouse nor any living children varies from 1 to 2% in the Republic of Korea and China, to 10 to 11% in Switzerland and Ireland, with the United States around 7% (39), which would suggest substantial differences in bereavement multipliers. It is also likely that the results would vary more dramatically if COVID-19 mortality followed alternative age gradients: For example, if deaths were concentrated among children. Part of the reason the results are so consistent for COVID-19 is that we focus on close relations, and due to the concentration of most deaths among grandparents, parents, and spouses, there are limits on the potential for bereavement. That is, the maximum number of spouses and biological parents and grandparents that could be lost are one, two, and four; few will lose a child, and almost none will lose a grandchild to COVID-19. In situations where mortality concentrates among the young, however, these caps would not apply and there would be substantial heterogeneity in mortality experiences, between people and potentially between epidemiological realities (3).

To test the sensitivity of our main results, which used estimates of infection fatality ratios from Wuhan, China that adjust for nonreporting and censoring (38), *SI Appendix*, Table S1, *Section C* shows what happens if we use unadjusted case fatality ratios from Italy (40). Note that the Italian case fatality ratios are substantially higher than the infection fatality ratios we focus on in the main text (leading in our simulations to more than twice as many deaths with approximately the same number of infections), as they do not account for asymptomatic and untested individuals, among other things. Despite this, our results are highly stable to the application of the Italian ratios, which shows that the bereavement multiplier will be 8.6 times the total death toll, within the range of our main text estimates. Supplementary analysis of the age distribution and kin loss distribution (*SI Appendix*, Figs. S1 and S2) using the Italian case fatality ratios show that these too are comparable to the main text results.

Although reliance on different infection fatality ratios only minorly shifts the estimated bereavement multipliers, it is plausible that regardless of the final infection fatality ratios, infection prevalence will not be uniform across the United States population. Just weeks into the American epidemic, state-level data confirms that Black Americans are disproportionately affected by COVID-19 (41). These disparities, combined with differences in the size and shape of kin networks between Black and White Americans, could mean that Blacks suffer more kin deaths than Whites, which could elevate the overall, combined bereavement multiplier for the United States population. *SI Appendix*, Table S1, *Section D* presents estimates for the whole United States population that assume a substantially higher burden of COVID-19 mortality among Black Americans, while *SI Appendix*, Figs. S3 and S4 show age and kin loss distributions under the same scenario. Specifically, given the lack of data on race-specific infection fatality ratios, we reestimated the bereavement multiplier assuming 10% of the United States White population becomes infected, and 50% of the Black population, which translates to substantially higher death prevalence among the latter group. As shown, such a scenario would push the total COVID-19 bereavement multiplier up to 9.3 times the death toll, again within the range we estimate in our main results. *SI Appendix*, Table S1, *Section F* presents race-specific multipliers while *SI Appendix*, Figs. S8–S10 show age- and kin-loss–specific estimates separately by race, which are generally in line with the population-averaged ones.

Just as it is plausible that the impact of COVID-19 may be unequally distributed across racial groups in America, it is also possible that the infection prevalence over the course of the epidemic will not be constant by age, but instead variable across age groups, which may also affect estimates of total kin loss. In *SI Appendix*, Figs. S5 and S6 and Table S1, *Section E*, we introduce an age-graded infection scenario that mimics estimates from Germany, Iceland, and other countries with broader access to testing (42). This scenario has lower infection burdens among young children and older adults and higher ones among middle-aged adults and leads to about 12% of the total population becoming infected (*SI Appendix*, *SI Narrative*). As shown, even different age patterns of infection produce only slight differences in the bereavement multiplier that are within the range we estimate in our main text results.

Although we demonstrate that our results are highly stable under different infection prevalence scenarios, because of the clear age gradient in COVID-19 mortality and the nature of kinship relations in the contemporary United States, the validity of other assumptions will influence the accuracy of our estimates. We would see differences if the age curve of infection fatality ratios were to differ substantially from the ones we examined, for example. Kin structures in America vary geographically, and the intensity of COVID-19 mortality is also likely to be geographically patterned. However, if infection becomes more widespread throughout the population, as many epidemiologists anticipate, geographic heterogeneity will decline over the course of the epidemic. Our results are also based on modeled approximations of the kinship structure of White and Black Americans, albeit ones that correspond well to empirical estimates (*SI Appendix*, Figs. S11–S14). Because of data limitations and the requirements of demographic microsimulation, we were unable to model the kinship networks of racial and ethnic groups other than White and Black Americans; unmodeled groups together comprise about 10.1% of the United States population (43). Finally, we cannot account for issues like living with or far from a person who dies, or whether people have strong or weak relationships with those who die, and we did not examine bereavement of step-relations, nonmarital partners, or other types of important kin (44, 45). Our estimates are aggregated, but of course there will be individual heterogeneity in how many kin are affected by COVID-19 mortality, with some of those who die leaving many and some leaving no family behind (46, 47).

## Discussion

Demographic science can help to understand the epidemiological patterning and mortality impacts of COVID-19 (48), but it can also shed light on important downstream social ramifications of this crisis. It will take many months, if not longer, to know how severe the mortality impacts of COVID-19 will be in terms of total numbers of deaths it causes in the United States and elsewhere; absent post hoc statistical models, contemporaneous records will undercount deaths from COVID-19 because not all deaths are accurately classified. Despite this current information deficit, today we can use demographic tools to project the extent to which COVID-19 mortality will reverberate across kin structures in America. Under various epidemiological scenarios, our analyses show that the burden of family bereavement from COVID-19 deaths will be higher than the COVID-19 death toll by nearly an order-of-magnitude: Each associated death will leave roughly nine times as many Americans bereaved by the death of a grandparent, parent, sibling, spouse, or child. That is, if 190,000 Americans die from COVID-19, as some models project by August 2020 (49), this will correspond with ∼1.7 million Americans having lost a grandparent, parent, sibling, spouse, or child due to COVID-19. If, for example, 1,000,000 eventually die from COVID-19 over a longer period, then 8.9 million would be bereaved, representing roughly 3 of every 100 Americans.

The scale at which COVID-19 mortality will lead to kin loss among surviving Americans suggests that COVID-19 might create a second wave of population health challenges tied to bereavement and the loss of social and economic support. An extensive literature demonstrates that, after experiencing the death of a close relation, individuals are at elevated risk of a host of negative life-course stressors, poorer health, and relationship strain (7, 10, 21–24). The vast scale of COVID-19 bereavement has the potential to lower educational achievement among youth, disrupt marriages, and lead to poorer physical and mental health across all age groups. The loss of kin ties will also limit important sources of social support, such as when grandparents provide childcare or siblings help one another manage older adult loneliness. Future research should be careful to include family bereavement as a possible life course antecedent to adverse outcomes across multiple life domains and stages.

It is also possible that the anticipated negative effects of the death of a family member on survivors may be even more severe given the unusually challenging and traumatizing circumstances surrounding family loss due to COVID-19 (50). First, deaths due to COVID-19 are sudden and unanticipated, which contrasts with the more protracted experience of losing a relative to a prolonged sickness that families in America typically experience. Sudden death can make it difficult to feel closure and can further complicate grieving (51). Second, the infectious nature of COVID-19 may preclude in-person interaction with ailing relatives (50). Instead, family members are physically separated from one another, unable to provide care and comfort, which can further contribute to intense grief (52). Third, COVID-19 is disrupting families’ ability to engage in traditional postmortem ritualization and memorialization due to restrictions on travel and the size of funerals and memorials (50). Fourth, the potential for clustering of deaths within families due to COVID-19’s highly transmissible nature will lead some families to simultaneously experience the death of multiple family members, further complicating the grieving process (53–57). Together, these distinct facets of COVID-19 may mean that kin loss during this pandemic may be uniquely traumatizing and bear more severe and numerous consequences for the bereaved than is the case in the recent mortality landscape. That the bereavement burden is nine times the death toll clarifies the need for further research to seriously consider COVID-19 bereavement and its consequences.

Thinking beyond the five types of close kin we studied, the overall burden of bereavement from COVID-19 will be higher than our estimates. The bereavement multiplier would be substantially higher if broader kin ties are considered, including in-laws, aunts, uncles, cousins, and even more removed family members. Efforts to quantify and analyze the loss of more distal relations will better clarify the full reach of COVID-19 bereavement, including through computational models like those used here and also survey approaches (33, 58). Of course, each COVID-19 death will also leave friends, coworkers, and neighbors grieving. And, the broader healthcare, social, and economic crises associated with COVID-19 could indirectly lead to higher mortality due to otherwise unrelated causes of death (e.g., untreated chronic conditions, alcohol misuse, self-harm, domestic violence, and other factors). Thus, complementary efforts to quantify and understand the collective grief of the entire COVID-19 crisis are essential to fully appreciate the psychological imprint left on the population after the pandemic abates.

Beyond quantifying the bereavement burden to COVID-19, this study more generally offers a tool for thinking about the collective experience of mortality, and the toll of specific causes of death. One can easily extend the logic of our study to generate bereavement multipliers for other leading causes of death in the United States, including heart disease and cancer, as well as other ongoing mortality crises, including the opioid epidemic and gun violence. Expanding the breadth of these estimates to other countries dealing with the COVID-19 crisis is also a ripe topic for future work, as is expanding their depth by delving further into subnational differences by factors such as race and gender. Within a simulation framework, the approach we articulated can flexibly answer questions like how deaths averted translates to fewer bereaved, and how disparities in the mortality burden contribute to disparities in bereavement, insights that are broadly applicable in numerous circumstances.

By demonstrating the multiplicative impact of a mortality shock as it reverberates within and across families, the bereavement multiplier is a valuable measure that can expand our understandings of the social impacts of ongoing epidemics, like COVID-19, as well as long-standing leading causes of death. The flexibility of the bereavement multiplier to diverse epidemiological realities enables researchers to track the growing family bereavement burden in lockstep with the death toll.

## Approach, Methods, Data, and Measures

Our analysis of bereavement multipliers draws on estimated kinship networks and population structures for White and Black Americans that were created with demographic microsimulation and have been analyzed in prior work (1, 2). Microsimulation is the most popular approach for understanding demographic contributions to kinship networks (59–61). It works by computationally modeling the actions of a synthetic population of individuals over time, allowing them to marry, divorce, give birth, and die according to specified age-, sex-, category-specific probabilities input by the researcher (with categories such as parity, marital status, or race). Briefly, the networks we drew on modeled both marital and nonmarital fertility, were “closed” microsimulation models wherein all participants other than the initial pool were born into the simulation (62), were constructed using the Socsim demographic microsimulation data (63), and were parameterized with age-, sex-, race-, and category- (marital status, parity) specific fertility, mortality, and marriage data from historical estimates and future projections (1). We focused on the subset of living individuals from the simulated July 2020 period in these data. Note that we conducted all simulations separately by race and created a combined file that weights the results to the White (85.1%) and Black (14.9%) percentages of the total United States population of single-race White and Black Americans (43).

Note that our simulation did not assign people to different types of living arrangements, and thus we were unable to estimate, for example, household-level experiences of bereavement or the role of group homes or other institutions. Unfortunately, because of the lack of household information in the simulations, we were not able to test intriguing hypotheses about intrahousehold transmission (48, 64, 65). Higher levels of household- or family-based transmission dynamics would lower bereavement multipliers by increasing multiple bereavement experiences (where deaths cluster within families); such patterns may increase racial disparities in multiple bereavements in line with race differences in multigenerational living (66). We note, however, that early contact tracing estimates seem to suggest relatively low rates of intrahousehold transmission (67).

Using these simulated data, we assigned each living individual a uniform random number between 0 and 1. In the main text, we then considered those whose assigned numbers were less than the infection prevalence threshold (e.g., 20% in the main text) as infected. We then assigned each of those who are infected a second uniform random number between 0 and 1 and compared this number to the infection fatality ratio associated with the given person’s age group. In the main text, we drew these infection fatality ratios from estimates that account for censoring, demography, and under-ascertainment in Wuhan, China (38). We considered those whose second random number was less than the infection fatality ratio associated with their age group to have died. The total number of deaths in the scenario formed the denominator of the bereavement multiplier. The numerator of the bereavement multiplier was the number of people who, through the kinship network data referenced above, we estimated were connected through grandparent, parent, sibling, spouse, or child ties to at least one person whom we considered as having died. We estimated age-specific and kin-type-specific bereavement multipliers in the same fashion, except that we limited the calculations of the numerator to those in the age group in question, those who lost at least one family member through the kin type in question, or both (we kept the denominator, deaths, constant across these population subgroups). Note that we repeated these processes 40 times to obtain estimates of variation. Our primary results are the medians of these distributions; we also present the upper and lower points that contain 95% of the distribution of simulated results.

The data we examined are derived from a demographic microsimulation, which contains numerous assumptions and approximations and are not measures of “real” kinship structures in the United States today. That said, the simulated kinship network data accurately replicate key features of real kinship networks in the United States. In supplemental analyses, we show the correspondence between the simulated kin data we use and weighted estimates of kin ties that account for kin undercoverage (37), which we made from the Panel Study of Income Dynamics’s Family Relationship Matrix data (*SI Appendix*, Figs. S11–S14). In particular, we examined the mean number of kin of different types and ages for focal respondents of different ages; for example, we compared the mean number of grandparents aged 80 to 89 y for people aged 20 to 29 y in the simulation data and as estimated from the Panel Study of Income Dynamics. See *SI Appendix, SI Narrative* for more details. These estimates show close correspondence between the simulated data and the Panel Study of Income Dynamics data in both the mean numbers of kin and the age patterning of ties.

We also tested alternate scenarios, including one that examines different infection prevalence scenarios (*SI Appendix*, Table S1, *Section B*), one that examines different infection fatality rates (*SI Appendix*, Figs. S1 and S2 and Table S1, *Section C*), one that examines differential distributions of death by race (*SI Appendix*, Figs. S3 and S4 and Table S1, *Section D*), and one that examines differential infection prevalence by age (*SI Appendix*, Figs. S5 and S6 and Table S1, *Section E*); each of these analyses were conducted in an analogous fashion to what we describe here. We also describe race-specific multipliers in different scenarios (*SI Appendix*, Figs. S7–S10 and Table S1, *Section F*,).

We provide data and code to replicate our findings in Dataset S1.

## Supplementary Material

Supplementary File

Supplementary File
