# Supplementary material for: Tracking the reach of COVID-19 kin loss with a bereavement multiplier applied to the United States
Source: Proc Natl Acad Sci U S A. 2020 Jul 10;117(30):17695–701. doi: 10.1073/pnas.2007476117 (PMC7395491; doi:10.1073/pnas.2007476117)
Supplement: Supplementary File [file pnas.2007476117.sapp.pdf]

SI Narrative

<u>Overview</u>

SI Table 1 and *SI Figures 1 to 10* examine the sensitivity of the bereavement multiplier estimates to the data source for the infection fatality ratios (IFRs), different assumptions about race and age differences in infection likelihoods, and race specific estimates of the multiples in the different scenarios. *SI Figures 11 to 14* examine the validity of the simulated kinship network data by comparing them to an empirical source of U.S. kinship data for Black and White residents.

1. <u>Main results in paper: Medians and 95% of distribution of estimates [in brackets]</u>

Section A at the top of *SI Table 1* reports the main results from Table 1 of the paper. It shows the median estimates of bereavement multipliers for the combined White and Black US population using adjusted estimates of Wuhan-based infection fatality ratios (IFRs) (1) and a 20% infection scenario uniformly distributed in the population (also presented in main text Table 1) as well as the upper and lower points in the distribution of estimates that contain 95% of our simulated results (the latter are in brackets).

2. <u>Sensitivity to Different Infection Prevalence Scenarios (*SI Table 1, Section B*).</u>

Section B examines how sensitive the estimates of the bereavement multipliers are to different infection prevalence scenarios. In the main text, we focus on a scenario where 20% of the population is infected uniformly at random. In this section, we examine what happens when 10% and 40% are infected uniformly at random. The results show the median estimates in the distribution of simulated outcomes. The multipliers are nearly identical across these scenarios and the ones we obtained from the 10% and 40% infection prevalence scenarios are squarely in the middle of the distribution of simulated outcomes we obtained in the main results (see bracketed values in Section A).

3. <u>Sensitivity Analysis for Infection Fatality Ratios from Italy (*SI Table 1, Section C*; *SI Figures 1-2*).</u>

Section C examines how sensitive the estimates of the bereavement multipliers are to application of different IFRs; in this case, estimates of case fatality ratios (CFRs) from Italy (2), which have not been adjusted for non-reporting and censoring and thus are considerably higher than the IFRs used in the main text (note that we treat these CFRs as IFRs in this simulation). Section C can be examined in comparison to Section A's main results. The results are similar across the two sources of mortality data, with the bereavement multiplier estimates 8.84 (main results) and 8.6 (Italian CFRs). The results in this section are clearly within the range of estimates we obtained in the main results (see bracketed values in Section A).

SI Figure 1 shows the age pattern of the bereavement burden for the estimates in Section C using the Italian IFR estimates. Note this is comparable to Figure 1 in the main text which uses the Chinese IFR estimates. The two figures show the same age pattern of bereavement with two peaks at ages 20-29 and 60-69. *SI Figure 2* presents the age pattern of bereavement by the type

www.pnas.org/cgi/doi/10.1073/pnas.2007476117

of kin lost using the Italian IFR estimates. Note that this is the equivalent of Figure 2 in the main text which draws on the Chinese IFR estimates. The two figures are remarkably similar.

4. Sensitivity to Non-uniform Infection by Race (*SI Table 1*, *Section D*; *SI Figures 2-4*)

Next, we examine the stability of the bereavement multiplier estimates if the burden of COVID-19 mortality is non-uniform across Black and White Americans. Section D of SI Table 1 presents estimates that assume a substantially higher burden of COVID-19 among Black Americans. Specifically, we re-estimated the bereavement multiplier assuming only 10% of the U.S. White population becomes infected, but 50% of the Black population. As shown, this would push the bereavement multiplier for the entire U.S. population up from 8.84 (under a 20% infection prevalence, see section A) to 9.3 times the death toll. These results are within the range of simulated outcomes we display in the main text (see bracketed results in Section A). The age pattern of this scenario is presented in *SI Figure 3*, and the age pattern by type of kin in *SI Figure 4*. These figures can be compared with Figures 1 and 2 in the main text.

5. Sensitivity to the Age Pattern of Infection Scenario (*SI Table 1*, *Section E*; *SI Figures 5-6*).

Just as it is plausible that the impact of COVID-19 may be unequally distributed across racial groups in America, the infection prevalence may also not be uniform across age groups; variable infection by age could affect estimates of total kin loss. *SI Table 1*, *Section E* examines bereavement multipliers for an alternative age-graded infection scenario, which mimics estimates from Germany, Iceland, and other countries with broader access to testing (3), as follows: 5% among 0-9 year olds, 10% among 10-19 year olds, 12.5% among 20-29 year olds, 15% among 30-39 year olds, 17.5% among 40-49 year olds, 15% among 50-59 year olds, 12.5% among 60-69 year olds, 10% among 70-79 year olds, and 5% among those aged 80 and above. Because of population structure, about 12% of the total population ends up infected in this scenario. As shown, assuming a lower burden of infection among young children, and a relatively higher one among middle age adults, produces a bereavement multiplier of 9.3, which is still within the range of simulated outcomes shown in the main text (see bracketed results in section A. *SI Figures 5 and 6* present the age pattern of bereavement for losing any type of kin (*SI Figure 5*) and age pattern of bereavement by kin type (*SI Figure 6*). These figures are the equivalent of main text Figures 1 and 2 respectively.

6. Variation in the Results by Race (*SI Table 1*, *Section F*; *Figures 7-10*).

Next, in Section F, we disaggregate the results for Whites and Blacks. We disaggregated both the main text results (the uniform 20% infection prevalence scenario shown in Section A) and the scenario with race differences in infection prevalence (10% for Whites, 50% for Blacks shown in section D). These results show similar stability in the bereavement multipliers, even among different race groups. For instance, compared to the aggregated results in section A, the race-specific multipliers for Whites in the same scenario are within the range of simulated outcomes (see bracketed results in Section A). For Blacks, the multipliers in this same scenario are slightly larger in total and for grandparent and parent bereavement, and they are slightly lower for sibling and spouse bereavement. These results are shown in *SI Figure 7* (age pattern of bereavement) and *SI Figure 8* (age pattern of bereavement by kin type). Looking at the results from the

scenarios where we model different infection prevalence by race, the results are again comparable (last two rows of *Table SI1*, *Section F*). These are visually depicted in *SI Figure 9* (age pattern) and *SI Figure 10* (age pattern by kin type).

7. Comparison of Simulated Kinship Networks to Panel Study of Income Dynamics (*SI Figures 11-14*)

Because our primary analyses are based on simulated kinship networks of Black and White Americans, a key question is how well these simulations capture the true distribution of kin relations and ego-alter ages, where ego is the focal person and alter their relative (i.e., when looking at how many 60 year old parents of 30 year olds there are, for instance, ego would be the 30 year old child and alter would be the 60 year old parent). As a comparison point, we rely on the Panel Study of Income Dynamics (https://psidonline.isr.umich.edu/), which began in 1968 as a conventional household survey, but which has since followed members of these households as they founded new households, effectively tracing the kinship networks of members of these lineages. Prior work has analyzed these data to create kinship networks (4, 5). This dataset has significant limitations, however. First, we rely on study non-response codes to infer when relatives have died rather than mortality linkages. Second, from the perspective of younger kin, only one half of their extended family is covered by these linkages – that of the PSID family. To address these issues, we assume that the kin count distribution in PSID and non-PSID families are equal on average, and therefore double all estimated grandparent counts (the only affected tie examined here) (4). Third, this dataset relies on household co-residence or chains thereof to identify interpersonal linkages – thus, kin who have never co-resided in the lineage, such as non-co-resident fathers, will not be identified in these data. Nonetheless, this data set provides the best available reference point for our simulations for the population of Black and White Americans today.

We estimated ego- and alter-age-specific kin counts for spouses, parents, grandparents, and siblings, using the PSID’s Family Relationships Matrix file. Egos were included if they were observed in one or more tie in the 2017 wave of the PSID. Alters were included if they were ever recorded as being connected to ego through one of the four relationships examined and were determined to be alive in 2017 according to PSID non-response codes. To account for kin under-coverage in the Panel Study of Income Dynamics, we double grandparent counts in all age ranges (4) and subset analyses to individuals for whom a grandparent was ever observed in the data for grandparent counts, and for whom a parent was ever observed for parent and sibling counts. Only spouses observed as current spouses in 2017 were included. All estimates are weighted using ego cross-sectional weights. The analytical file does not distinguish between biological and adoptive parent/child and sibling ties, but it does distinguish these ties from stepparent and sibling ties.

SI Figures 11-14 show comparisons of the age-specific kin counts observed in the PSID and the kinship simulation. Generally, the simulation captures the PSID distribution of ego- and alter-age-specific kin counts quite well when there is adequate sample size in the PSID (at ego ages younger than 70-79). The fit is excellent for spouses (*SI Figure 14*), as both graphs show high degrees of age homophily between ego and alter for spousal pairs. The fit is similarly excellent for grandparents (*SI Figure 11*), with both simulated and PSID results showing expected

intergenerational spacing patterns. For parents (*SI Figure 12*), the PSID estimates are generally slightly lower than those predicted by the simulation model. This is an expected outcome, due to the PSID's reliance on household co-residence to identify parent-child linkages – a limitation that does not apply to the simulated data. For sibling data (*SI Figure 13*), the fit is excellent for alters in their 50s, but poorer at older ages, reflecting small sample sizes in the PSID in these age ranges (stemming from the restriction to individuals for whom the PSID ever observed parents, typically coresident ones, meaning that few egos 70 and older are observed that meet restriction since the survey began in 1968). All together, while the simulation data are an approximation, they provide key features of kinship networks that are quite close to what is observed in empirical data.

8. References for SI

1. R. Verity, *et al.*, Estimates of the severity of coronavirus disease 2019: a model-based analysis. *The Lancet Infectious Diseases* (2020).

2. E. Livingston, K. Bucher, Coronavirus disease 2019 (COVID-19) in Italy. *Jama* (2020).

3. D. F. Gudbjartsson, *et al.*, Early Spread of SARS-Cov-2 in the Icelandic Population. *medRxiv* (2020).

4. J. Daw, A. M. Verdery, R. Margolis, Kin count (s): Educational and racial differences in extended kinship in the United States. *Population and development review* **42**, 491 (2016).

5. J. Daw, A. M. Verdery, S. E. Patterson, Beyond household walls: the spatial structure of American extended kinship networks. *Mathematical Population Studies* **26**, 208–237 (2019).

9. SI Figures and Tables

SI Figure 1. The age pattern of the bereavement burden in the scenario using Italian CFRs in place of the IFRs used in the main text: Bereavement multipliers for losing any type of kin by age group by infection prevalence scenario. Note: Kin types included in the bereavement burden are grandparent, parent, sibling, spouse, and child. The area under the curve sums to the "any kin" bereavement multipliers in SI Table 1, Section C. Compare to Main Text Figure 1.

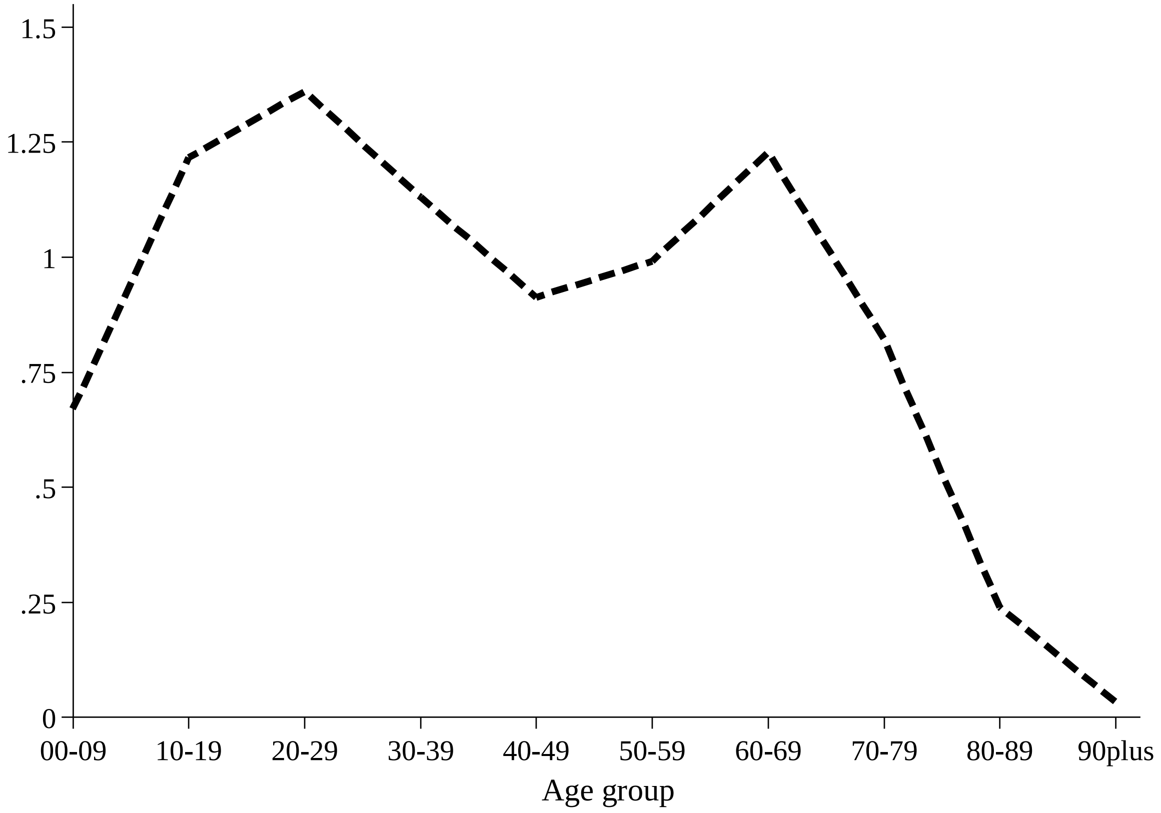

SI Figure 2. The age pattern of bereavement burden by type of kin death in the scenario using Italian CFRs in place of the IFRs used in the main text. The areas under each curve sum to the named kin bereavement multipliers in SI Table 1, Section C. Compare to Main Text Figure 2.

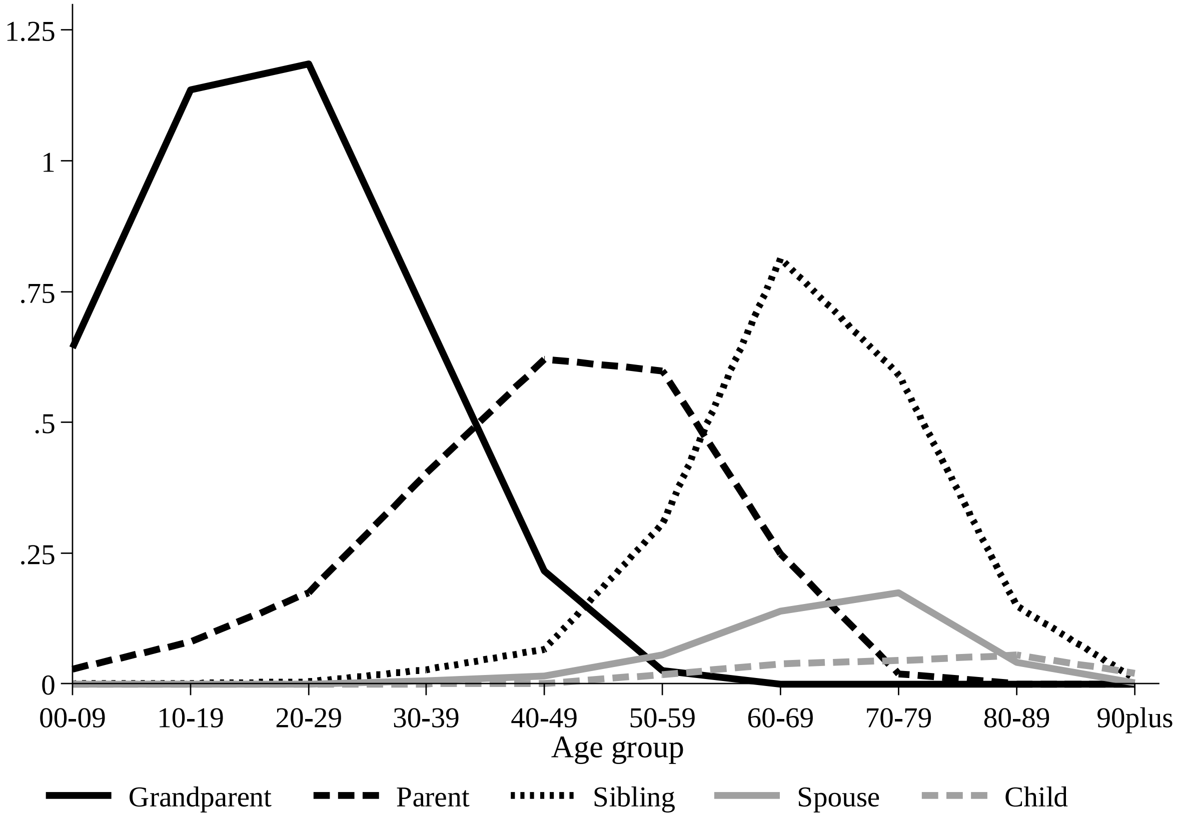

SI Figure 3. The age pattern of the bereavement burden in the scenario using differential infections by race: Bereavement multipliers for death of any type of kin by age group by infection prevalence scenario. Note: Kin types included in the bereavement burden are grandparent, parent, sibling, spouse, and child. The area under the curve sums to the "any kin" bereavement multipliers in SI Table 1, Section D. Compare to Main Text Figure 1.

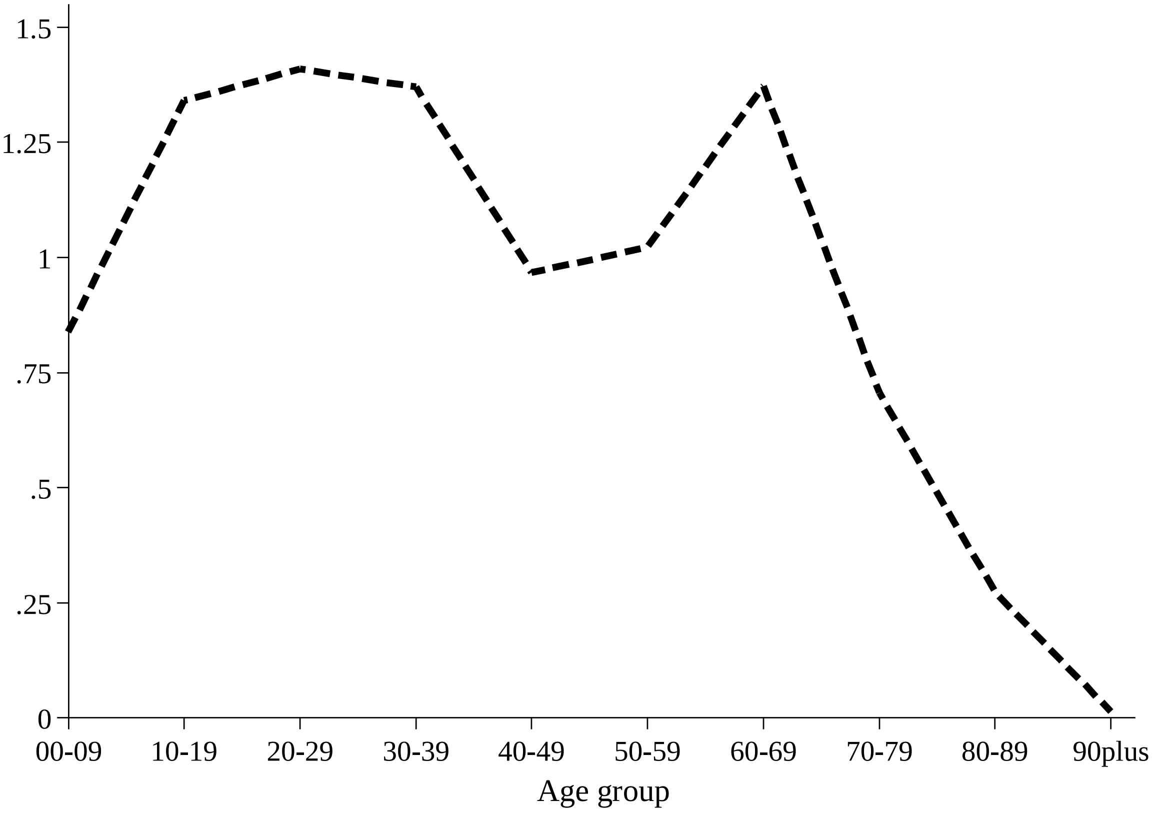

SI Figure 4. The age pattern of bereavement burden by type of kin death in the scenario using differential infections by race. The areas under each curve sum to the named kin bereavement multipliers in SI Table 1, Section D. Compare to Main Text Figure 2.

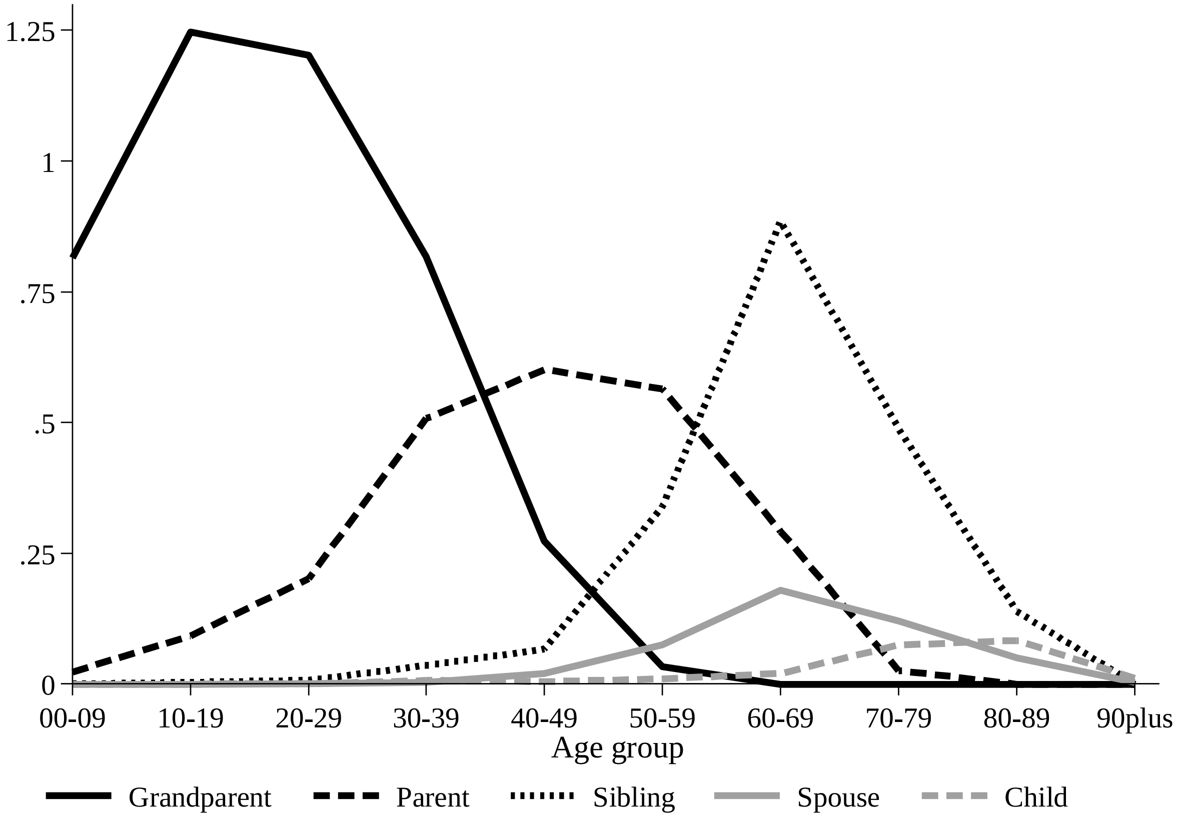

SI Figure 5. The age pattern of the bereavement burden in the scenario using differential infections by age: Bereavement multipliers for death of any type of kin by age group by infection prevalence scenario. Note: Kin types included in the bereavement burden are grandparent, parent, sibling, spouse, and child. The area under the curve sums to the "any kin" bereavement multipliers in SI Table 1, Section E. Compare to Main Text Figure 1.

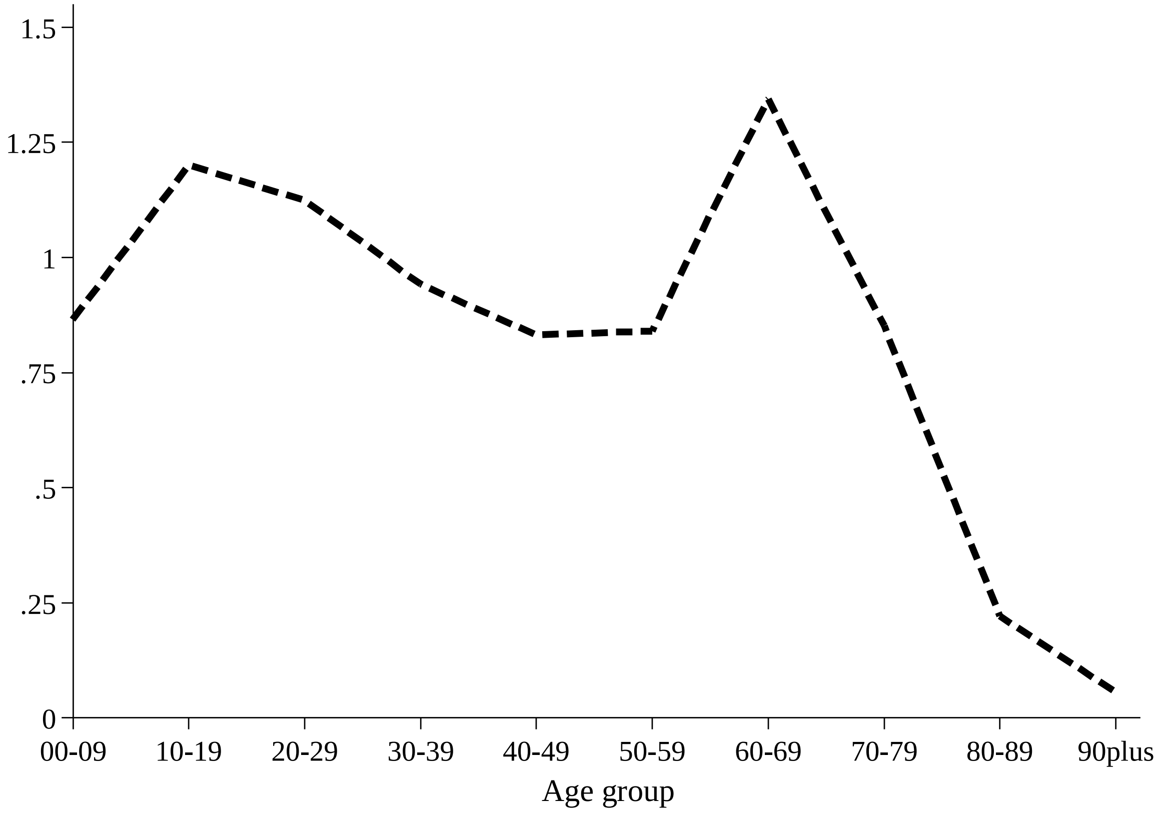

SI Figure 6. The age pattern of bereavement burden by type of kin death in the scenario using differential infections by age. The areas under each curve sum to the named kin bereavement multipliers in SI Table 1, Section E. Compare to Main Text Figure 2.

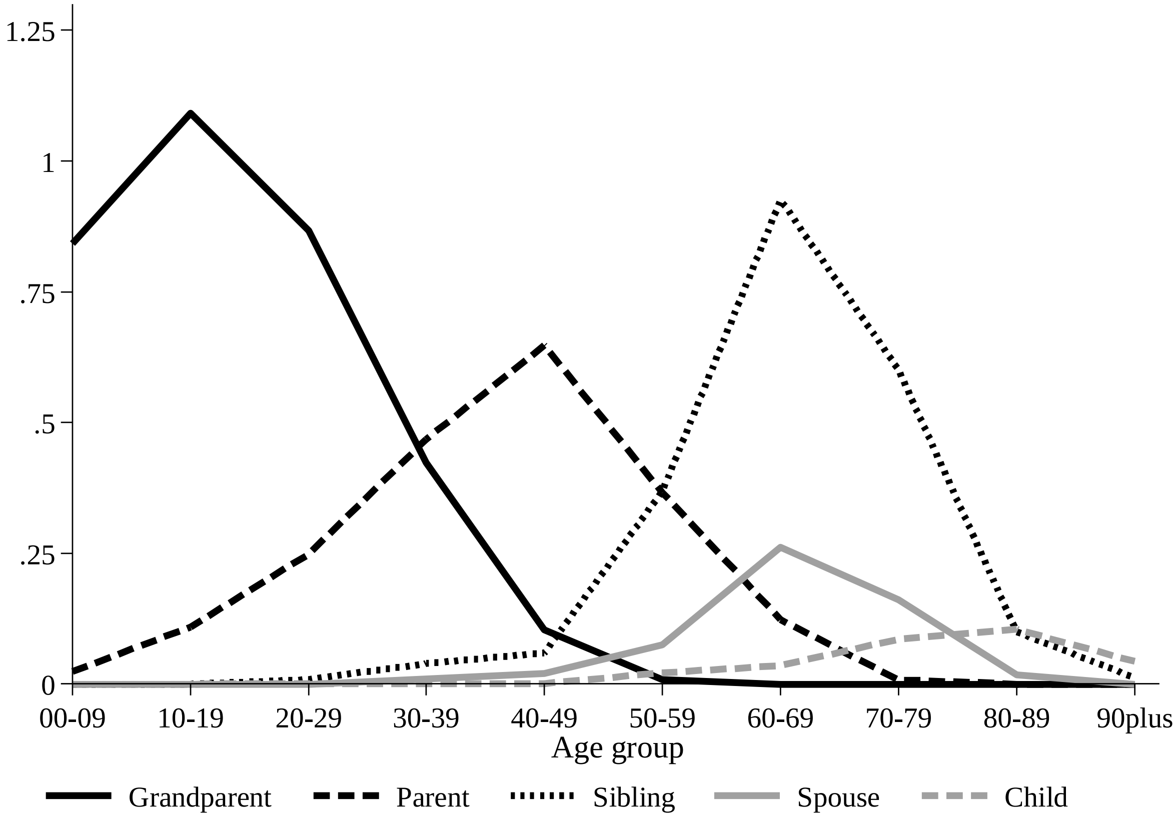

SI Figure 7. Race-specific age patterns of the bereavement burden in the main text results: Bereavement multipliers for death of any type of kin by age group by infection prevalence scenario. Note: Kin types included in the bereavement burden are grandparent, parent, sibling, spouse, and child. The area under the curve sums to the "any kin" bereavement multipliers in SI Table 1, Section F. Compare to Main Text Figure 1.

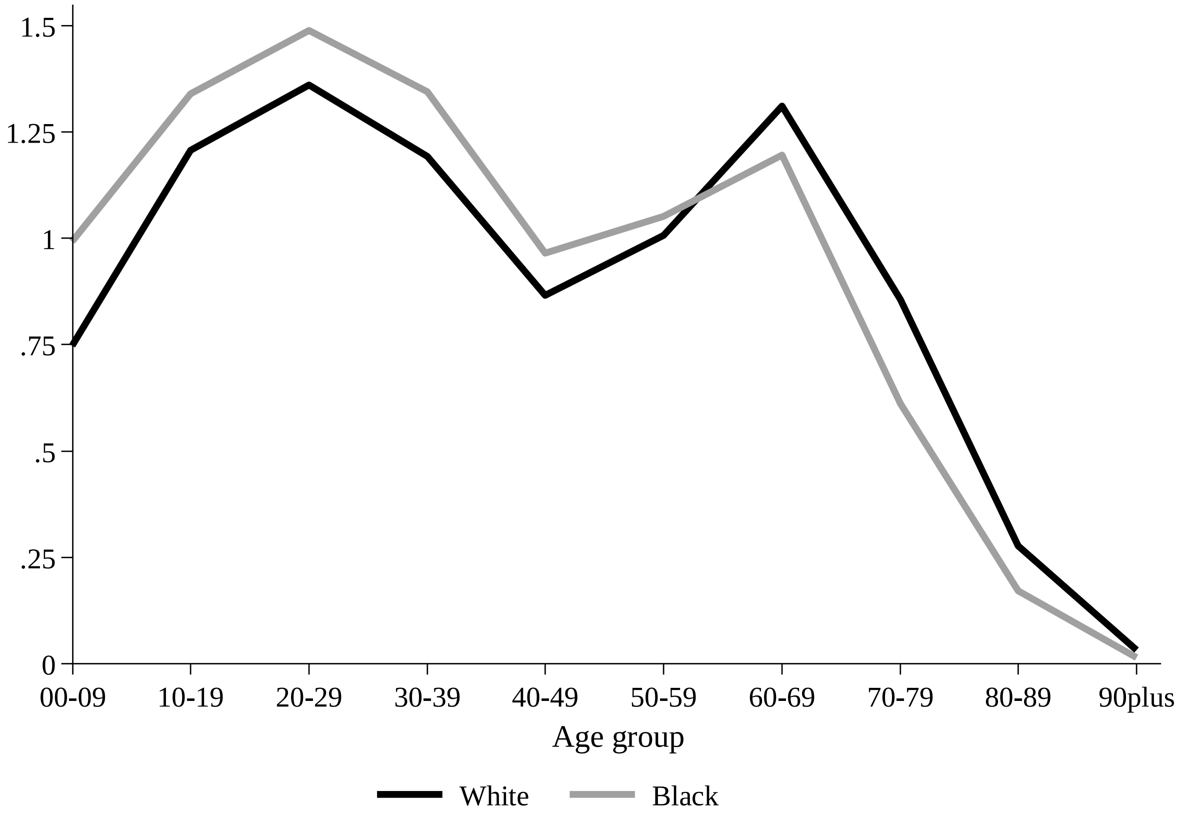

SI Figure 8. Race-specific age patterns of bereavement burden by type of kin death in the main text results. The areas under each curve sum to the named kin bereavement multipliers in SI Table 1, Section F. Compare to Main Text Figure 2.

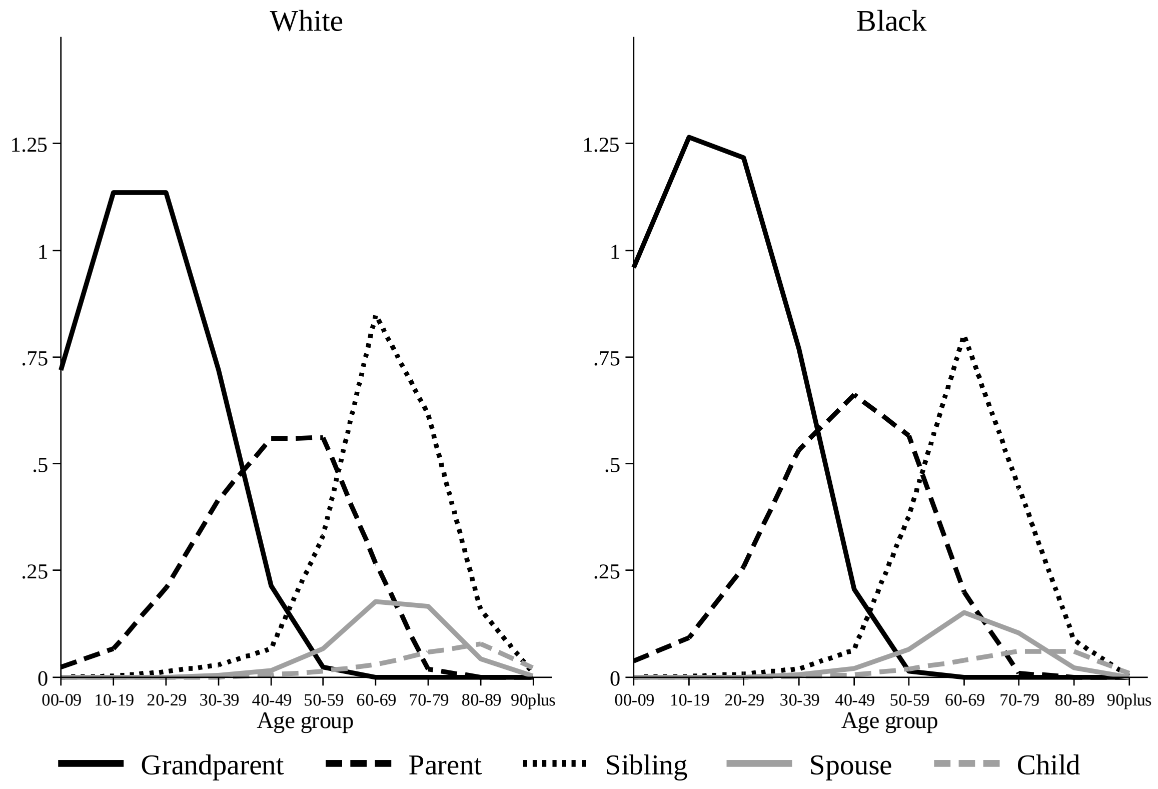

SI Figure 9. Race-specific age patterns of the bereavement burden in the scenario using differential infections by race: Bereavement multipliers for death of any type of kin by age group by infection prevalence scenario. Note: Kin types included in the bereavement burden are grandparent, parent, sibling, spouse, and child. The area under the curve sums to the "any kin" bereavement multipliers in SI Table 1, Section F. Compare to Main Text Figure 1.

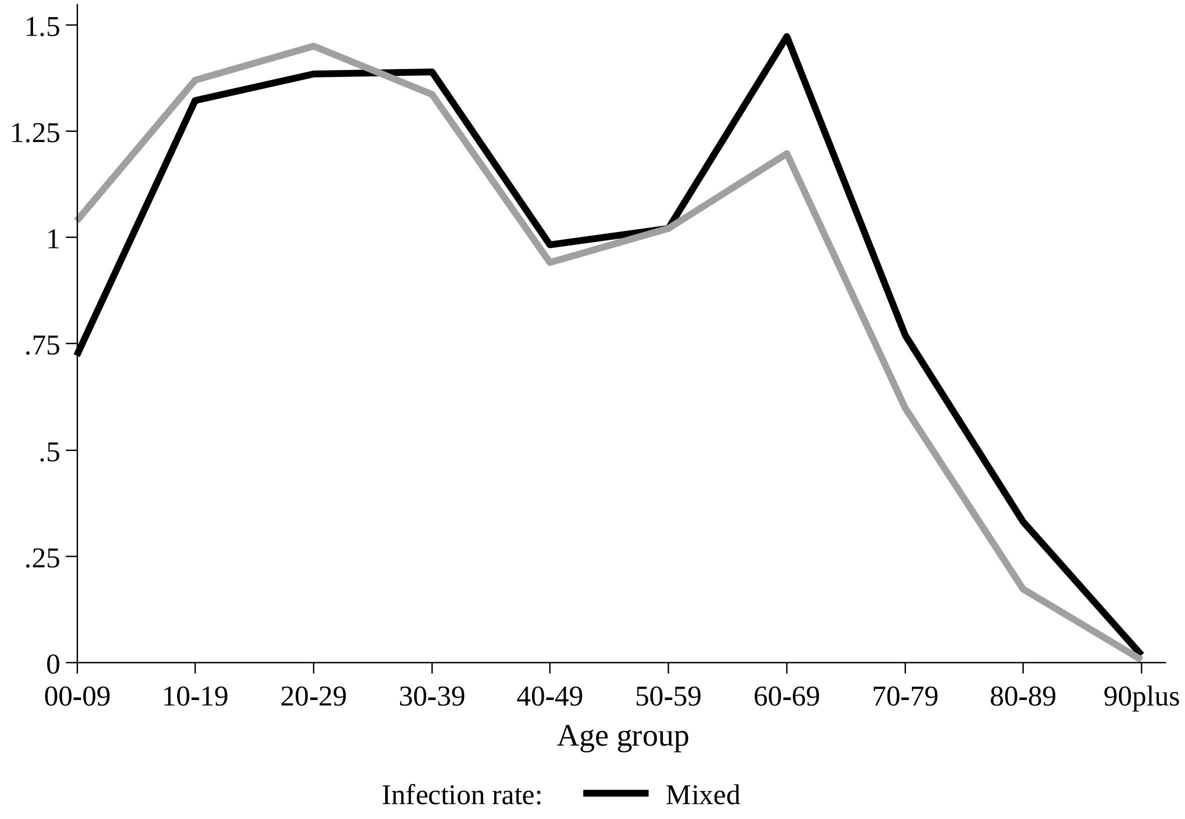

SI Figure 10. Race-specific age patterns of bereavement burden by type of kin death in the scenario using differential infections by race. The areas under each curve sum to the named kin bereavement multipliers in SI Table 1, Section F. Compare to Main Text Figure 2.

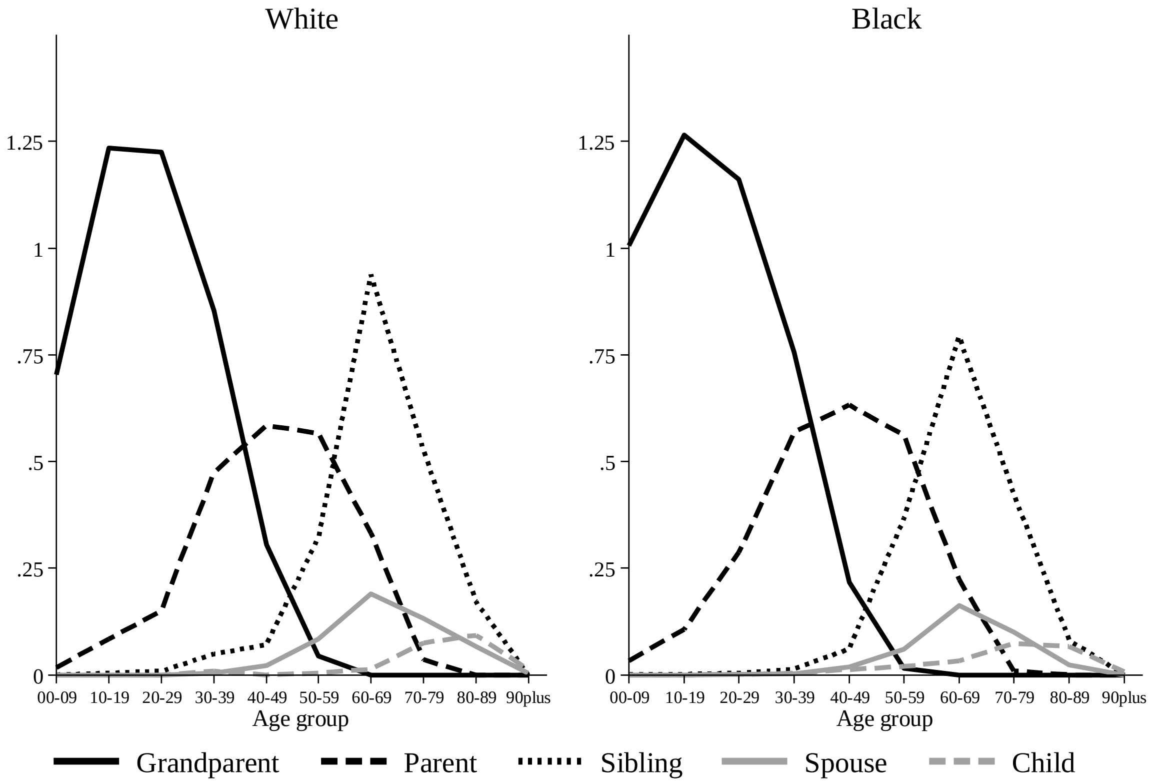

SI Figure 11. Comparison of the age patterning of grandparent ties in the simulation results and the Panel Study of Income Dynamics.

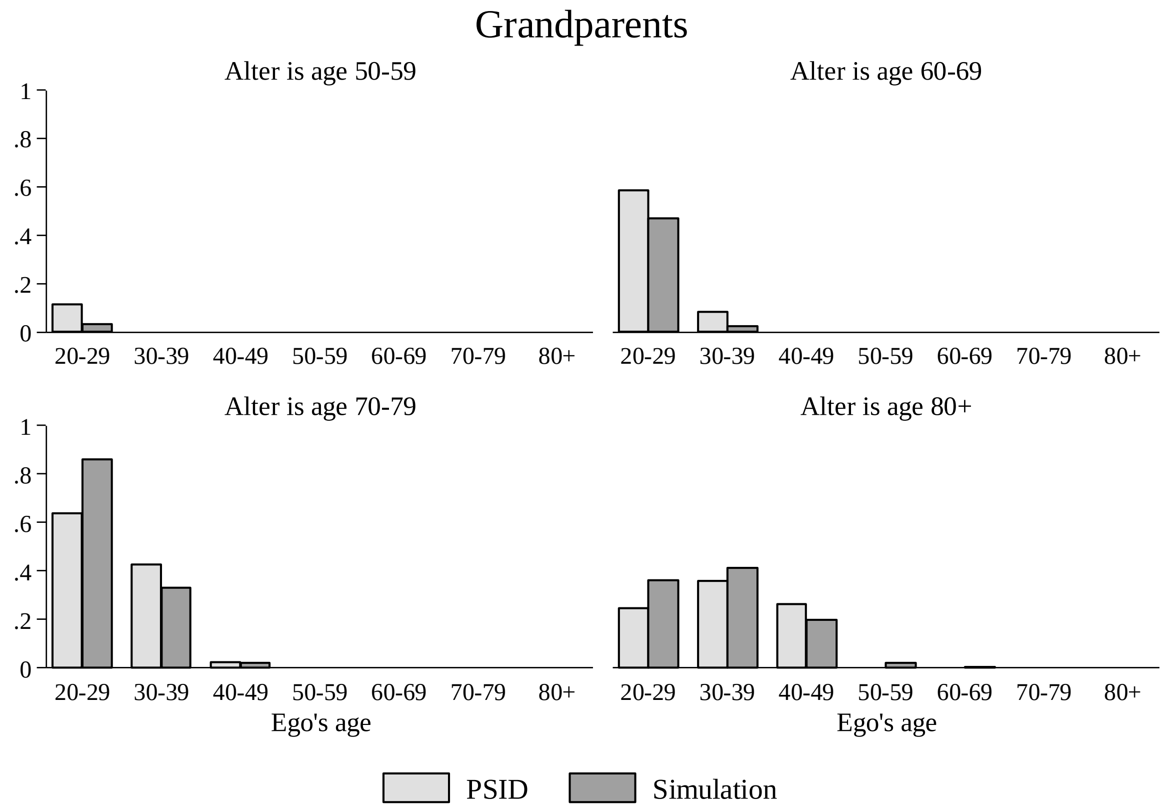

SI Figure 12. Comparison of the age patterning of parent ties in the simulation results and the Panel Study of Income Dynamics.

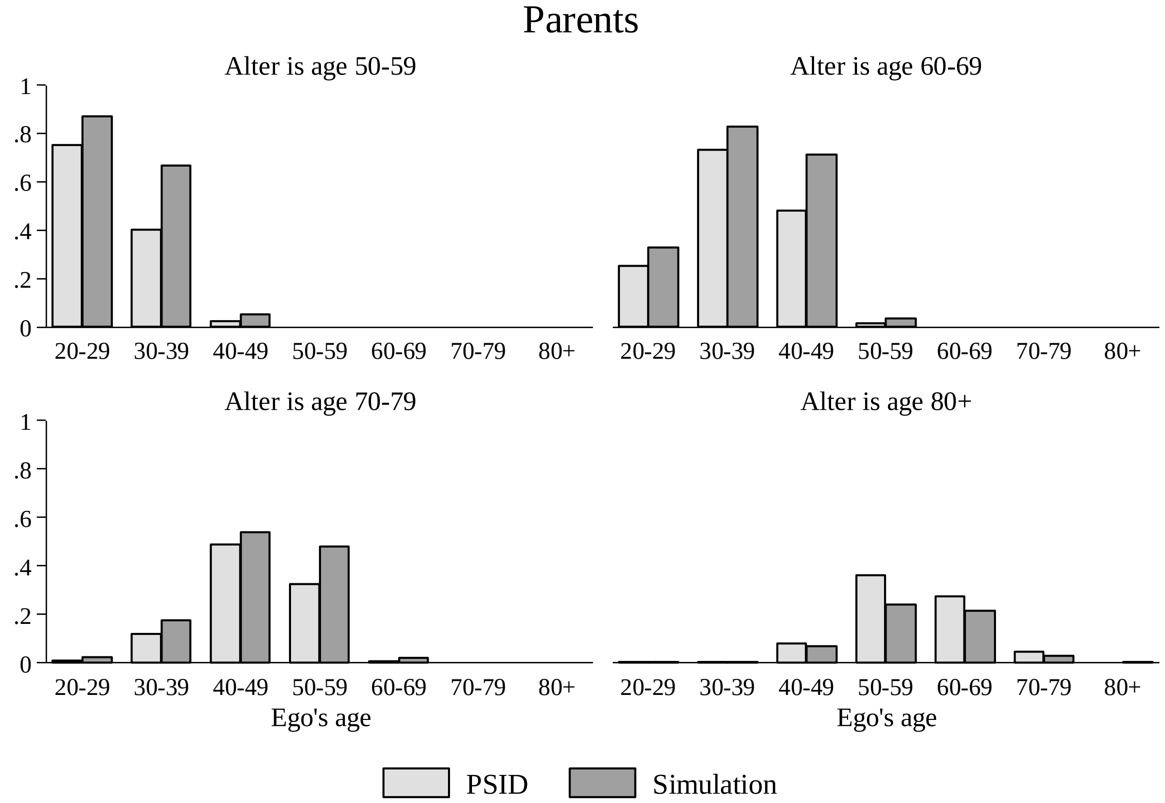

SI Figure 13. Comparison of the age patterning of sibling ties in the simulation results and the Panel Study of Income Dynamics.

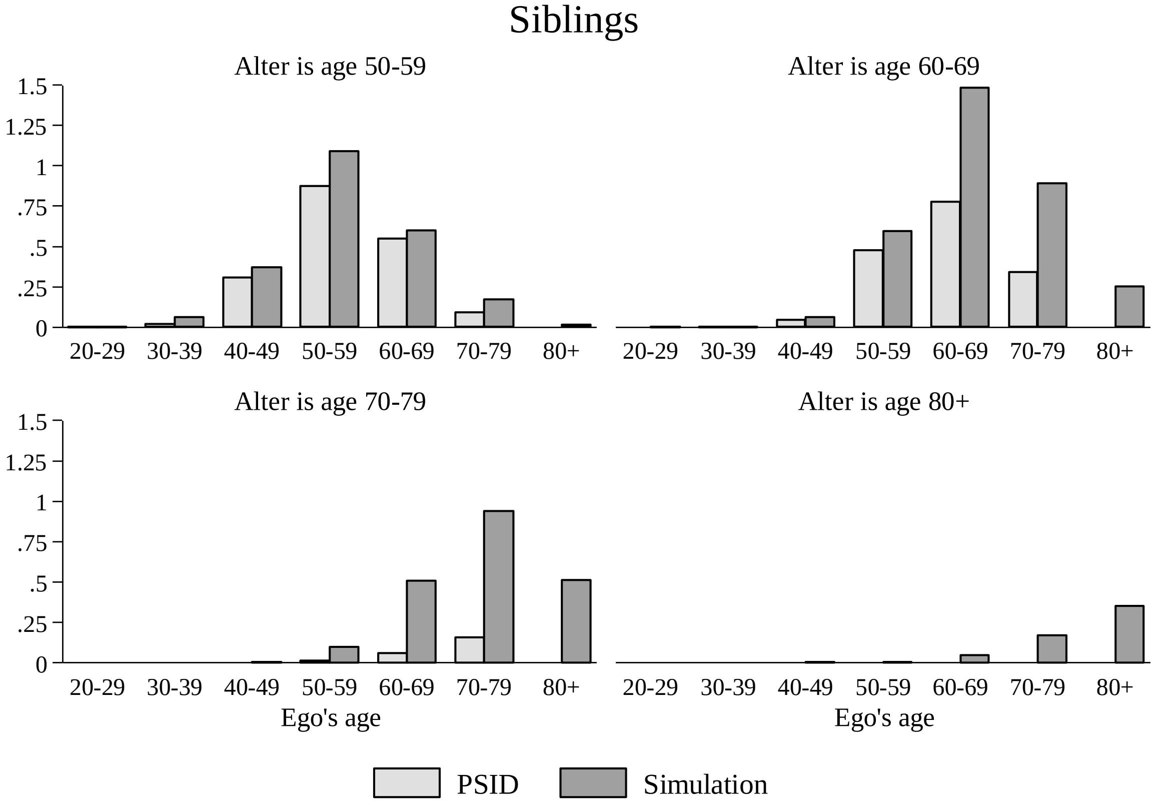

SI Figure 14. Comparison of the age patterning of spouse ties in the simulation results and the Panel Study of Income Dynamics.

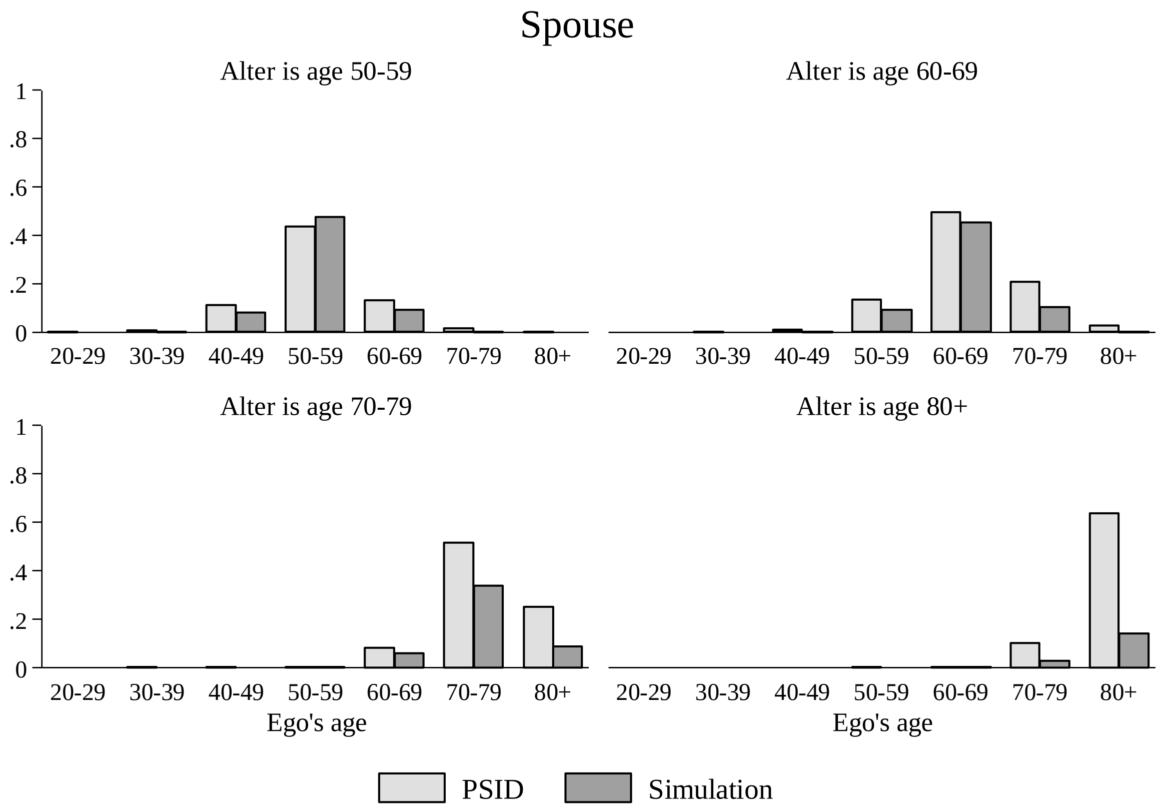

SI Table 1. Comparison of bereavement multipliers for various infection scenarios.

Infection Scenario				Bereavement multiplier[1]: Number of people who would lose the named kin for each death					
**Percent of population w/ simulated infection**	**Source of IFR data**	**Modeling of infection risk**	**Race group to which applies**	**Any type[2]**	**Grandparent**	**Parent**	**Sibling**	**Spouse**	**Child**
Section A: Main results in paper: Medians and 95% of distribution of estimates [in brackets]									
20%	Wuhan, China	Uniformly distributed	Whites and Blacks	8.9 [7.4,10.4]	4.0 [3.1,4.9]	2.2 [1.6,2.8]	2.0 [1.6,2.6]	0.5 [0.3,0.7]	0.2 [0.1,0.4]
Section B: Sensitivity to different infection prevalence scenarios									
10%	Wuhan, China	Uniformly distributed	Whites and Blacks	8.8	4.0	2.1	2.0	0.5	0.2
40%	Wuhan, China	Uniformly distributed	Whites and Blacks	8.8	4.0	2.3	2.0	0.5	0.2
Section C: Sensitivity to alternate age-specific mortality rates from Italy									
20%	Italy[3]	Uniformly distributed	Whites and Blacks	8.6	3.9	2.2	2.0	0.5	0.2
Section D: Sensitivity to non-uniform infection by race									
10% Whites, 50% Blacks	Wuhan, China	Unevenly distributed by race[4]	Whites and Blacks	9.3	4.4	2.3	2.0	0.4	0.2
Section E: Sensitivity to non-uniform infection by age									
12%[5]	Wuhan, China	Unevenly distributed by age	Whites and Blacks	8.3	3.3	2.0	2.1	0.6	0.3
Section F: Variation in the results by race									
20%	Wuhan, China	Uniformly distributed (section A)	Whites	8.9	4.0	2.1	2.1	0.5	0.2
20%	Wuhan, China	Uniformly distributed (section A)	Blacks	9.2	4.4	2.4	1.8	0.4	0.2
10% of Whites	Wuhan, China	Unevenly distributed by race (section C)	Whites	9.4	4.0	2.2	2.1	0.5	0.2
50% of Blacks	Wuhan, China	Unevenly distributed by race (section C)	Blacks	9.1	4.4	2.4	1.7	0.3	0.2

Notes:
[1] A bereavement multiplier of 4 in the grandparent column means that if 100,000 people die, 400,000 grandchildren would lose at least one grandparent.
[2] Any type includes a grandparent, parent, sibling, spouse or child.
[3] The Italian data contains raw case fatality ratios, not infection fatality ratios (IFRs); they are thus substantially higher than the infection fatality ratios because they do not adjust for underestimation of cases and other factors. We treat these CFRs as IFRs in this sensitivity test.

[4] Modeling different infection prevalence by race under race-constant infection fatality rates will increase disparities in the burden of deaths; given the lack of race-specific infection fatality rate data, we took this approach.
[5] The age graded infection scenarios assume that infection prevalence varies by age in ways roughly consistent with documented results from Germany, Iceland, and other high-testing contexts, albeit with higher levels of total infection. Specifically, it assumes age risks of infection are: 5% among ages 0-9, 10% among ages 10-19, 12.5% among ages 20-29, 15% among ages 30-39, 17.5% among ages 40-49, 15% among ages 50-59, 12.5% among ages 60-69, 10% among ages 70-79, and 5% among ages 80 and above.
